# Effect of localized hypoxia on *Drosophila* embryo development

**DOI:** 10.1371/journal.pone.0185267

**Published:** 2017-09-21

**Authors:** Zhinan Wang, Shawn C. Oppegard, David T. Eddington, Jun Cheng

**Affiliations:** Department of Bioengineering, University of Illinois at Chicago, Chicago, Illinois, United States of America; University of Iceland, ICELAND

## Abstract

Environmental stress, such as oxygen deprivation, affects various cellular activities and developmental processes. In this study, we directly investigated *Drosophila* embryo development *in vivo* while cultured on a microfluidic device, which imposed an oxygen gradient on the developing embryos. The designed microfluidic device enabled both temporal and spatial control of the local oxygen gradient applied to the live embryos. Time-lapse live cell imaging was used to monitor the morphology and cellular migration patterns as embryos were placed in various geometries relative to the oxygen gradient. Results show that pole cell movement and tail retraction during *Drosophila* embryogenesis are highly sensitive to oxygen concentrations. Through modeling, we also estimated the oxygen permeability across the *Drosophila* embryonic layers for the first time using parameters measured on our oxygen control device.

## Introduction

Intercellular and extracellular oxygen levels are well known to affect many biological processes. The ability to sense and respond to various oxygen conditions is critical to maintain oxygen homeostasis at tissues level in mammals through a series of sophisticated physiological networks. Oxygen deprivation (e.g. hypoxia) leads to deleterious effects, such as slowing growth and affecting cellular development [1, 2]. *In vitro* studies have also shown that the oxygen levels can affect enzyme expression [3] and stem cell differentiation [4, 5]. Among many kinds of animal models in the hypoxia studies, *Drosophila melanogaster* is recognized as a powerful biological model, due to its rich genetic resources, to study the molecular pathways involved in oxygen sensing and the response to the environmental impact [6–10]. Using this model system, previous studies show environmental oxygen levels are closely related to adult *Drosophila* metabolic rate [10], cell size [11], feeding preferences [12], cardiac responses [13] and body mass [14]. Notably, due to its highly dynamic and well-characterized morphology changes during embryogenesis, *Drosophila* embryo is also widely used in the study of hypoxia related responses, including finding the physiological sensors of hypoxia, the cell cycle checkpoints mechanism that senses the surrounding oxygen level and the mediators of low oxygen levels during a cell cycle [6–8, 15]. These findings prove that the oxygen level was highly affecting the cell cycle kinetics and cell division in *Drosophila* embryo. These findings may be used as the keys to answer many questions raised from the investigation of how hypoxia alters organism development and its compensating mechanism. However, little has been studied on how oxygen deprivation on microscale may affect *Drosophila* embryogenesis at certain region where key developmental activities occur.

To further answer these questions, we developed a method of using microfluidic devices with a precise spatial and temporal control of oxygen condition to evaluate the hypoxic response of *Drosophila* embryo. Microfluidic technology can provide precise control and manipulation of fluid flow in channels on the scale of micrometers [16]. To date, microfluidic devices have found many biological applications, including DNA analysis and fragment separation [17–19], PCR amplification [20, 21], protein analysis and enzyme assays [22–24], cell culture, and sorting [25, 26]. Additionally, microfluidic devices can also generate a chemical and gaseous gradient on microscale resolution, providing a direct way to study oxygen-sensitive cellular behavior in their microenvironment at a high temporal and spatial resolution levels. Microfluidic devices made from gas permeable polydimethylsiloxane (PDMS) have been widely used to introduce oxygen gradient based on oxygen-nitrogen exchange and diffusion [27, 28]. Microfluidic devices have been increasingly used to study *Drosophila* embryo development in temperature [29] and morphogen gradients [30].

In this study, we visualized cellular morphology and migration during several embryonic developmental stages lively under a variety of oxygen conditions. Since the designed microfluidic device could provide well-controlled microscale oxygen conditions to the *Drosophila* embryos, we became interested in how a developmental activity might be affected by localized hypoxia and whether it was compensated by the activities at other body parts under different oxygen conditions. The results showed that two of the key embryo developments, the pole cell movement during germband extension and the tail retraction during germband shortening, were delayed under certain levels of oxygen deprivation. In addition, a free diffusion model was developed and the oxygen permeability across the embryonic layers was estimated for the first time. Our findings proved the availability and uniqueness of applying microfluidic device in the study of live tissue in hypoxia by introducing finely controlled oxygen conditions on microscale to specific regions in live tissue. Our methods also showed the potential of recognizing the regulators of the pathways in response to hypoxia at genetics level.

## Materials and methods

### Fly stock and husbandry

All fly stocks were raised on the standard Bloomington medium at 25°C. The following fly stocks were used: *Ubi- α-tubulin-GFP* [31] was used to visualize the pole cells; *UAS-mCD8-GFP;en-GAL4* was used to visualize the *engrailed* patterns.

### Embryo collection and preparation

The grape agar plates and *Drosophila* embryo collecting cages (Genesee Scientific) were used for the embryo collection. To prepare the grape agar plates, the mixture of grape agar powder (Genesee Scientific) and water were stirred and heated up. The mixture was then poured into the petri dishes and cooled down to room temperature for later use. Young (0–5 days old) flies were fed with yeast paste on the grape agar plate inside a collecting cage. A synchronized embryo collection was performed. Generally, the first two collections of the day were discarded and different waiting time was added to obtain the most embryos at the same desired developing stage.

The collected embryos were washed with the embryo washing solution (EWS, 7% NaCl and 0.03% Triton X-100, Sigma), and then dechorionated in 50% bleach (Clorox) plus 50% EWS for 3–5 minutes. Dechorionated embryos were finally rinsed with EWS thoroughly.

### Microfluidic device fabrication

The membrane and microchannel components were fabricated as described previously [32–34]. Briefly, a microchannel master was made using standard SU-8 photolithography and replica molding. PDMS was replica-molded on top of the SU-8 master at 75°C for 2 hours. To make the gas-permeable membrane, PDMS prepolymer was poured on top of a silicon wafer and spun to a thickness of 100 μm. The membrane was bonded to this frame using plasma surface activation, and then inlet/outlet holes were punched through both. This assembly was then aligned and bonded to the microchannel component.

The gas permeable membrane permits establishment of a concentration gradient between the infused gas in the microchannels below and the embryo culture volume above, driving diffusion to achieve a target steady-state oxygen concentration. The gas microchannel design consists of two separate channels that initially run parallel to one another and then wind cyclically to cover the rest of the device surface, yielding a sharp gradient of oxygen when two separate gases are infused.

### Embryo placement

**Fig 1:**
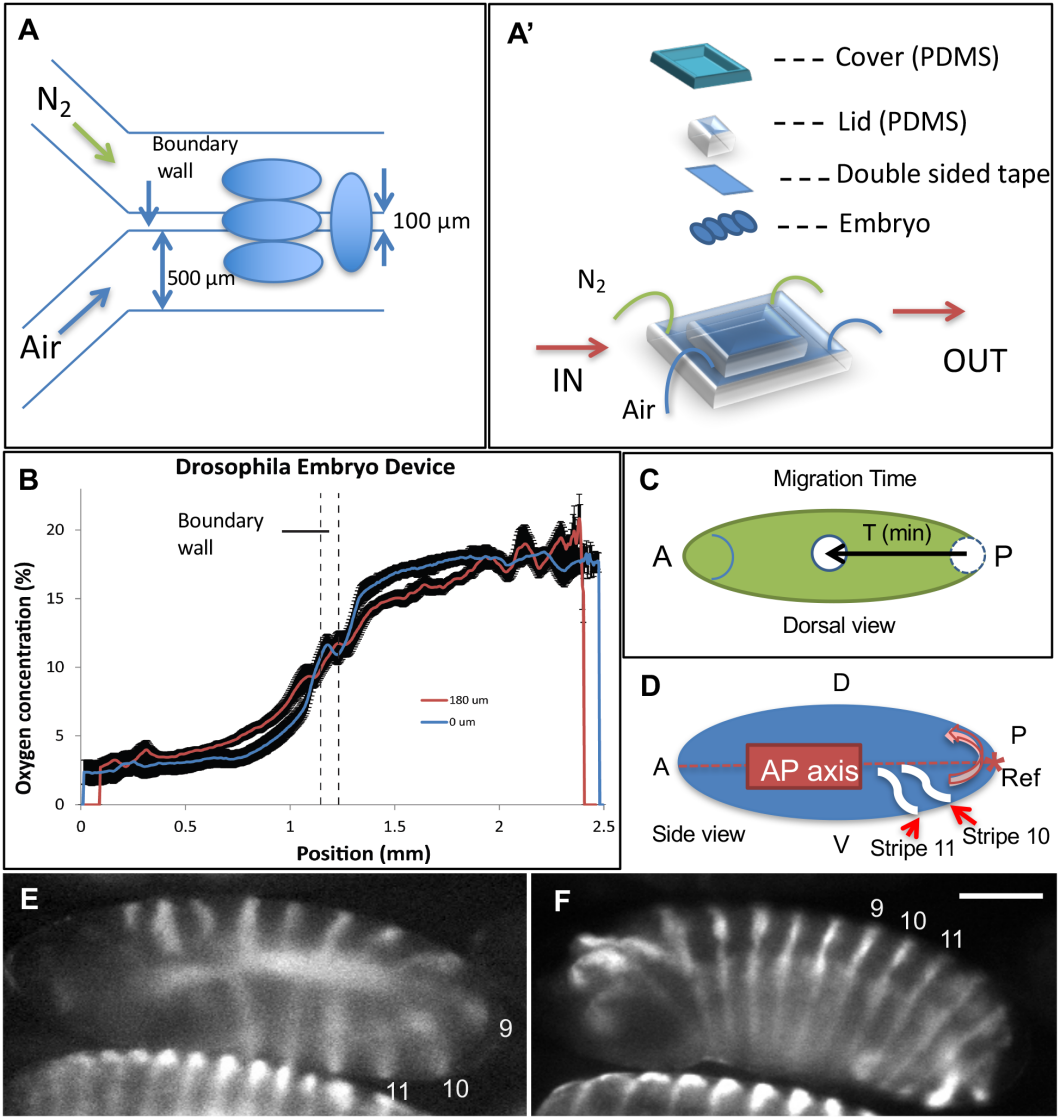
Experimental setup and the quantification methods. **(A and A’)** An illustration depicting the microfluidic channels and the positioning of the embryos in a microfluidic device, which is designed to establish the oxygen concentration gradients through microchannels with different infused gases. **(B)** The oxygen concentration profile inside the microfluidic device was measured. The oxygen concentration gradients at 0 μm and 180 μm above the gas emitting surface are plotted using 20 points rolling average, shown as blue and red lines, respectively. **(C)** The pole cell migration time was measured as the pole cells (white plate) move from 10% to 90% of their total migration distance. **(D)** An illustration shows two ventral stripes (10^th^ and 11^th^) pass through the posterior (reference point: *) of the embryo body with a counter-clockwise migration as indicated by the pink arrow. The time difference is defined as the *engrailed* stripe migration time. A: anterior; D: dorsal side; P: posterior; V: ventral side. Scale bar: 100 μm. **(E and F)** *engrailed* patterns (14 stripes) are shown before (E) and after (F) the tail retraction. The 9^th^-11^th^ stripes are labeled indicating the stripe migration.

Embryos can be positioned either perpendicular or parallel to the microfluidic channels. At different locations, the oxygen concentrations may vary, but are directly correlated to the distances measured from the center boundary wall. The oxygen concentration can be maintained and ranges from 3% to 20% on the entire microchannel surface. Particularly, to introduce the largest gas gradient from an embryo’s anterior to posterior, the embryos need to be positioned perpendicular to the channels and span the center boundary wall (Fig 1A and 1B). During imaging, the embryos were immersed in the media and immobilized on a double-sided tape with a PDMS lid. Last, a PDMS cover was applied on top to minimize media evaporation and finalize the imaging chamber (Fig 1A’).

### Oxygen concentration quantification

A platinum-based fluorescent oxygen sensor, PtOEPK (Frontier Scientific, Cat. No. O40969), was used to measure the oxygen concentration at room temperature. The PtOEPK dye (0.05% PtOEPK in polystyrene/toluene solution) was spin-coated on a glass coverslip, dried, and then peeled from the coverslip. The dye film was diced to the proper size to fit inside microfluidic device.

A fluorescence-equipped Olympus IX71 microscope, CCD camera (QImaging Retiga-SRV) and Metamorph image acquisition software (v7.6.3.0) were used for capturing images of the sensor. Data were taken for infusion of 0% and 21% oxygen into the two microchannels, with the sensor positioned both adjacent to the membrane (0 μm), and 180 μm above the membrane, which reflects the thickness of an embryo. All images were acquired at room temperature using a PtOEPK-compatible fluorescent filter with an excitation/emission of 595/760 nm and a 620 nm dichroic mirror (Chroma, VT). Images were taken with a 2× objective (PlanApo 2×/0.08). A 75W Xenon burner (Olympus U-LH75X) was used to illuminate the sensor. Images were analyzed for pixel intensity (Metamorph), and oxygen concentration was then calculated using the Stern-Volmer equation relating fluorescent intensity to oxygen concentration. Fig 1B depicts the capability of the device to create an oxygen concentration gradient.

### Live embryo fluorescence microscopy imaging

Embryos were placed at the specified locations inside the microfluidic device and imaged by an epifluorescence microscope (Zeiss Axio Observer Z1). The samples were illuminated by a fluorescence microscope light source (X-Cite 120Q). The time-lapse images were taken by a 10x objective (A-plan 10×/0.25) and captured by an EM-CCD camera (Hamamastu C9100-13). All images were processed and analyzed by Zeiss AxioVision and ImageJ.

### Embryo development quantification

#### Pole cells migration tracking and quantification

The pole cells in the fly embryos are a group of primordial germ cells that later become the gametes. During the formation of the syncytial blastoderm (2 hours after embryo is laid), a plate of approximately 150 pole cells initially form at the embryo posterior end, and then migrate anteriorly during the germ band extension [35]. When *α*-tubulin is ubiquitously marked by GFP, the pole cells can be identified with a stronger GFP signal at the posterior end, and their position can be quantified as the distance from the center of pole cell cluster to the posterior end. Taking the slight variation of the embryo size into consideration, the pole cell position was normalized by the embryo length and called the effective length (EL, 0<EL<1) thereafter. Hence, the average migration velocity of the pole cells has the unit of EL/min. The total pole cell migration time is quantified as the time duration of migrating from 10% to 90% of the overall migration distance (Fig 1C).

#### *engrailed* stripe migration tracking and quantification

*engrailed* is one of the primary segmentation genes required for the development of the posterior compartments of segments during embryogenesis [36, 37]. The gene expression of *engrailed* begins with the cellular blastoderm, so that 14 segmented stripes start to form [38] (Fig 1E). During the subsequent developmental stages, e.g. gastrulation, germband extension and germband shortening, all the 14 stripes migrate towards the anterior (Fig 1F). The *engrailed* stripe migration time hereby is defined as the time interval for two adjacent dorsal stripes (the 10^th^ and 11^th^ stripe) passing the reference point (posterior tip) (Fig 1D).

### Mathematical model of estimating the oxygen permeability across the embryonic layers

To simplify the discussion without losing generality, oxygen is assumed to diffuse freely and is consumed by all the embryo cells at the same time. We also assumed that the cells consume oxygen with the same rate, which is not affected by the oxygen deprivation when it occurs. In our model, finite elements (n>1000) were used to constitute a notional 3D *Drosophila* embryo. For each element that has the same shape (cube) and volume, we assumed that, at steady state, the mass of all oxygen that flows into it equals to the mass out of it, as well as the mass consumed by all the cells in each element:

$$\sum_{n=1}^{6}\left(C_{i\_n}-C_{i}\right)\cdot \eta \cdot S=RV$$ (1)

where

C_i_: the oxygen concentration of the element i, [mol/m^3^];

C_i_n_: the oxygen concentration outside the n^th^ surface of the element i, [mol/m^3^]. (n = 1,2,3…6);

S: the side area of the cube element, [m^2^];

V: the volume of the cube element, [m^3^];

R: the universal oxygen consumption rate in the embryo, [mol/m^3^];

*l*: the edge length of the cube element, [m];

D: the diffusivity of oxygen in embryo tissue, [m^2^/s];

E: the oxygen permeability through embryo outer layers [m/s];

*η*: the oxygen exchange parameter, which equals to D/*l*, when the element is inside the body; or E, when the element is on the periphery of the body;

Two of the major parameters, the oxygen diffusivity and the cellular oxygen consumption rate, were chosen based on earlier reports (see Table 1). Using MATLAB program (R2016B), the above equations were numerically solved for given parameters as shown in Table 1 and the oxygen profile inside the embryo (*C*_*i*_) was obtained. To evaluate the local oxygen concentration impact on the *engrailed* stripe migration speed precisely, only the portion (75% of the embryo body length), where the *engrailed* stripes locate, was used to calculate the average oxygen concentration.

**Table 1:** Parameters and values used in the simulation.

Symbol	Name	Unit	Value used in simulation	Reported number
D	Oxygen diffusivity	$\left[\frac{m^{2}}{s}\right]$	1.0×10^−9^	0.85×10^−9^ [39] 1.75×10^−9^[40] 1.0×10^−9^ [41]
*R*	Oxygen consumption rate	$\left[\frac{mol}{m^{3}\cdot s}\right]$	0.02	0.016 ^(1)^ 0.024 ^(2)^ 0.008–0.036 ^(3)^
*η*	Permeability of embryo	$\left[\frac{m}{s}\right]$	TBD	N.A

The parameters and their values used in the simulation are presented in the table. Note that the reported oxygen consumption rates listed above are converted based on the original values from the literatures using the sf9 cell line (*Spodoptera frugiperda*):

^(1)^ 0.15 μmol/10^6^cells/h [42, 43];

^(2)^ 0.22 mmol/10^9^cells/h [44];

^(3)^ 2.0–11.4 mmol/10^9^cells/day[45].

Embryo cell sizes were considered to convert cell numbers into volume, which is required in our modeling calculation. The oxygen consumption value for *Drosophila* embryo cells is chosen as 1/8 of the reported average sf9 cell line values considering their cell size difference.

### Statistics and data analysis

The P-values in all figures are calculated based on the student t-test using Microsoft Excel (14.4.6). All error bars indicate the standard deviation.

## Results

### Oxygen concentration gradient inside a microfluidic device

Firstly, the oxygen concentration was mapped out using the oxygen sensor calibrated to known oxygen concentrations (0%, 10%, and 21% oxygen, data not shown). The oxygen gradients at heights of 180 μm (the average embryo thickness) and 0 μm inside the microfluidic device were measured and plotted in Fig 1B. As expected from gas diffusion, the peak oxygen gradient at 0 μm height (0.028%/μm) is sharper than that (0.023%/μm) at 180 μm height. By understanding the oxygen gradient profile through the microfluidic device surface, we could better estimate the oxygen condition using location information, which is directly measurable from microscopic images.

### Delayed pole cell migration during embryogenesis

Upon cellularization at stage 5 during fly embryogenesis, the pole cells, also known as the precursor cells of primordial germ cells, initially form at the posterior end of a *Drosophila* embryo. During gastrulation, the future posterior midgut cells associated with pole cells start to migrate towards the center of the embryo body on the dorsal side [46–48]. Being one of the first major morphogenetic movements in the early gastrulation, pole cell migration was chosen as a good indicator of the midgut anlage invagination and germband extension [49]. We studied how it could be affected by hypoxia during early embryogenesis.

**Fig 2:**
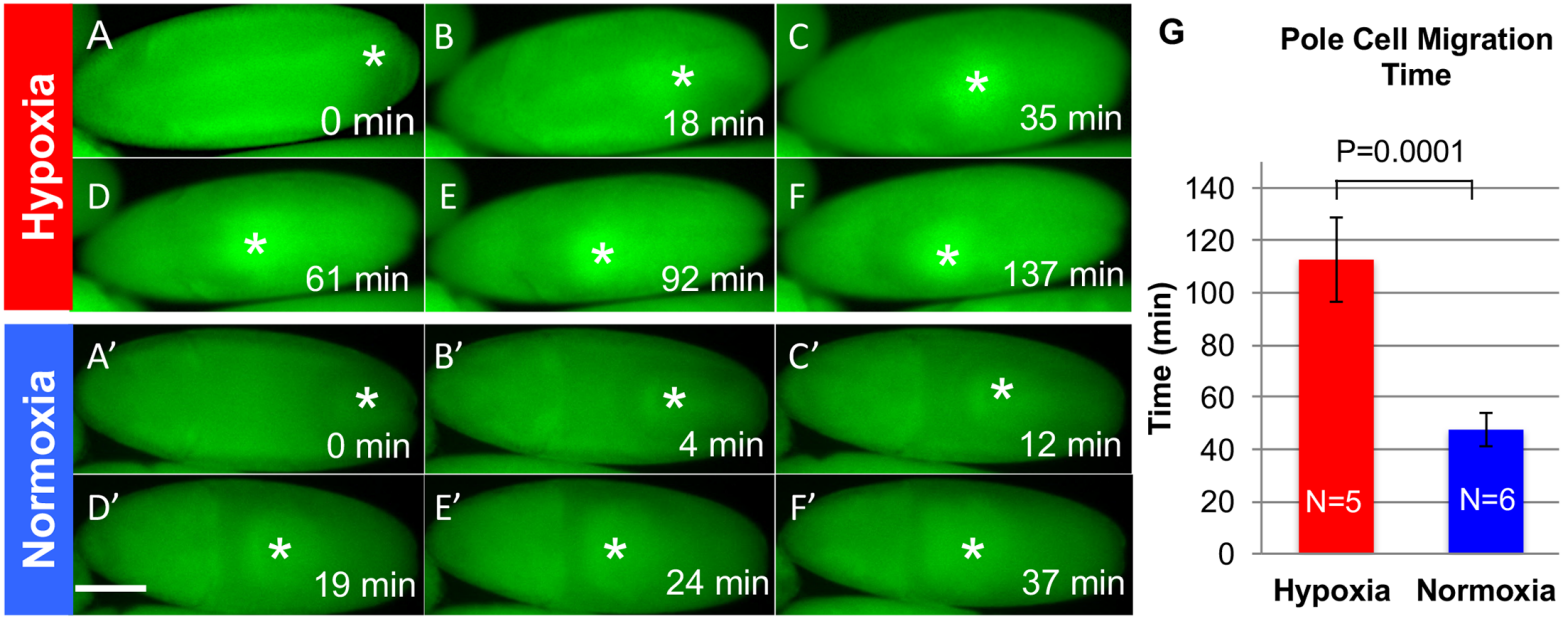
Pole cells move slower in hypoxia. Selected frames from a time-lapse analysis show α-tubulin green fluorescent protein (α-tub-GFP) *Drosophila* embryos are exposed to hypoxia **(A-F)** and normoxia **(A’-F’)**, respectively. The pole cells migration time is 37 min for a normoxic embryo and 137min for a hypoxic embryo. Stars indicate the center of pole cell clusters. Scale Bar: 100*μ*m. **(G)** Pole cells migration time under hypoxia and normoxia.

Two hours old embryos were placed at hypoxic ([O_2_] = 3%) and normoxic ([O_2_] = 20%) region in the microfluidic device with the anterior-posterior (AP) axis in parallel with the microchannels. We assumed the entire body of each embryo was under the same oxygen condition, since the oxygen concentration difference along an embryo’s ventral-dorsal (VD) axis was very small at these two regions. As shown in Fig 2A–2F, it took 37 minutes for the pole cells under a normoxic condition to migrate from the posterior end to the embryo center, while it took 137 minutes for the pole cells under a hypoxic condition to do so (Fig 2A’–2F’). Our overall results, on average, show a 237% slowdown of pole cell migration under hypoxia (112.6 minutes, n = 5) compared with under normoxia (47.5 minutes, n = 6), (Fig 2G). This is indicating that the pole cell migration can be delayed but not stopped at a sever oxygen deficiency (~%3).

### Delayed germ band shortening during embryogenesis

The germ band shortening occurs about 7 hours after the embryo is laid and lasts 2 hours by retracting the embryo tail from its previous location to the embryo’s posterior end. This is an essential step during the embryogenesis before the generation of stomatogastric neural system and major organ formation [35, 50]. The expression of the segment polarity gene *engrailed* is directly regulated by a group of pair-rule genes, such as *even-skipped* and *fushi tarazu* [37, 51], resulting in a pattern that consists of 14 stripes. The *engrailed* stripes initially appear during the cellular blastoderm stage (3 hours after egg is laid) [38] and distribute almost evenly along the outer layers of the embryo (Fig 1E). Subsequently, during the germ band shortening, *engrailed* stripes on the ventral side migrate toward the dorsal side via the posterior end resulting in the pattern shown in Fig 1F. Due to this severe morphological change, these stripes were used as the convenient markers to visualize and quantify the dynamics of the germ band shortening process in embryos exposed to various oxygen conditions.

To study how the germ band shortening was affected by the environmental oxygen condition and the compensating mechanism, *en-Gal4;UAS-a-tub-GFP* flies were generated to visualize the *engrailed* stripes in live embryos. The embryos were collected 5 hours after they were laid. They were positioned at different locations with the AP axis parallel with the microchannels. The distance between the AP axis of each embryo and the center wall was measured to estimate the oxygen condition based on the oxygen gradient profile shown in Fig 1B. Here, we assumed that the anterior and posterior of each embryo are under the same oxygen condition. The *engrailed* stripe migration time was quantified as the time interval of the 10^th^ and the 11^th^ stripe migrating over the embryo posterior (ref. point in Fig 1D).

**Fig 3:**
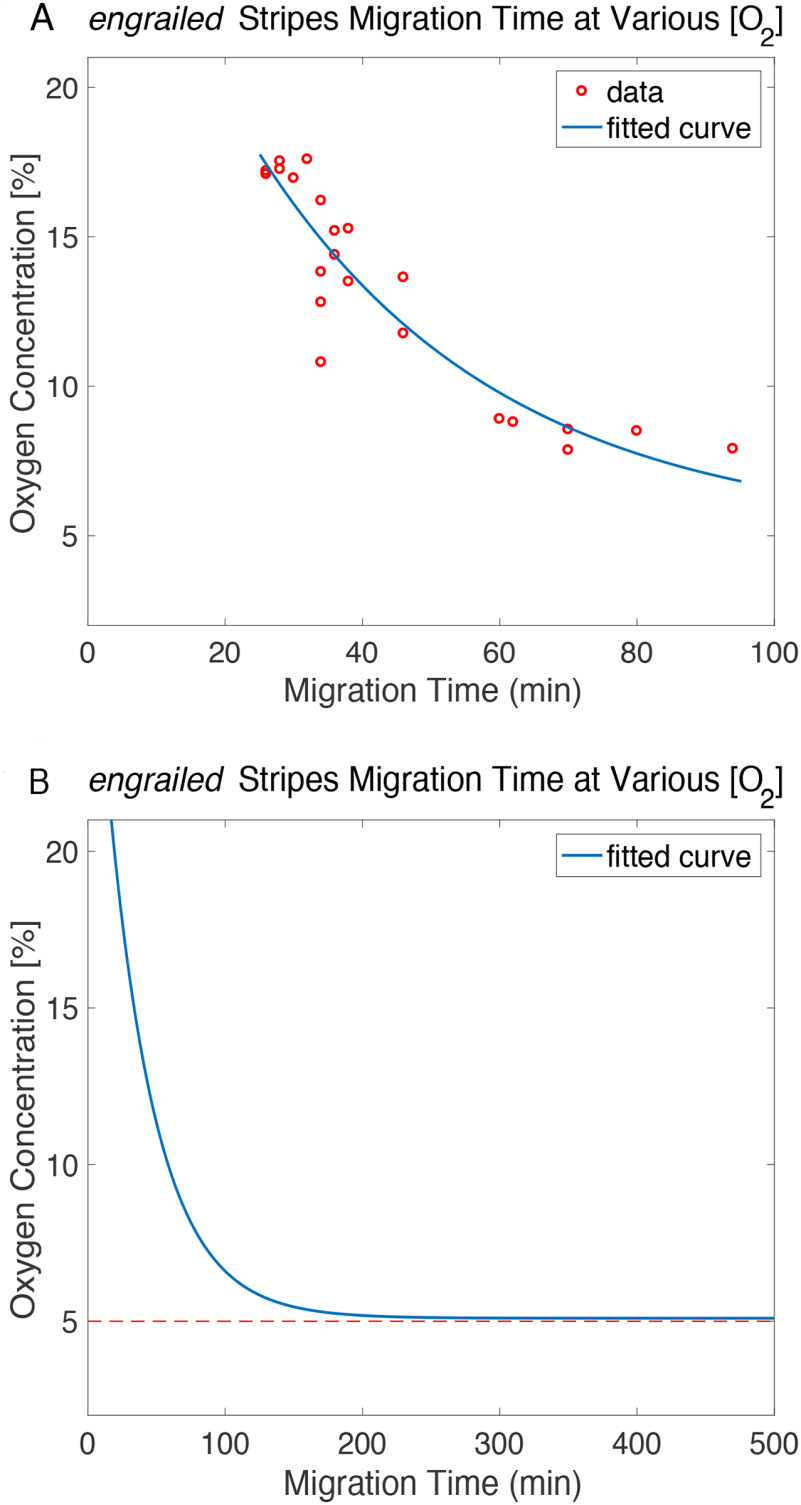
*engrailed* stripe migration time under different oxygen levels. **(A)** Data (red circles) showed every *engrailed* stripe migration time that measured at various oxygen conditions. The best-fit (least squares) curve (blue line) showed the delay on *engrailed* migration time when oxygen concentration dropped. **(B)** The extended best-fit curve (blue), combined with a horizontal asymptote line (dashed), showed the trend of the delay and the critical condition at 4.9% of oxygen, which could be the cutoff oxygen level that paused the germband shortening process.

From the tracking of the *engrailed* stripe migration, we saw a unique trend of time delay as the oxygen concentration decreased (Fig 3A). Firstly, we noticed that close to the normoxia, a slightly lower oxygen level did not affect *engrailed* stripe migration significantly: when compared with the migration time (22 ± 2 min) in normoxia, a slightly longer migration time (28 ± 2 min) was seen from the embryos at positions where the oxygen concentration is about 16% or higher (see S1 Movie). As the oxygen concentration dropped, e.g. from 16% to 10%, the stripe migration time increased to 38 ± 4 min. However, at the region where the oxygen concentration is even lower, e.g. between 9% and 7%, the effect of insufficient oxygen supply on the tail retraction was more revealing: the average migration time could be sharply increased by 50% from 60 min to 92 min due to a 2% drop on the oxygen concentration. The extreme cases were found at the position where the oxygen concentration was lower than 5%: the *engrailed* stripe migration did not occur in any of the imaging sessions (n = 5) that lasted 500 min or longer.

Our initial presumption was the migration time, as an oxygen sensitive probe, was following an exponential decay as the oxygen concentration dropped. To quantitatively understand this trend, we fitted our experimental data with an adapted one-term exponential curve that explained our findings (Fig 3B). The best-fit curve (least squares line) can be expressed as:

$$\left[{\text{O}}_{2}\right]=4.93+2480\cdot \text{exp}\left[-0.03(t\;+\;167.3)\right]$$ (2)

where, t is the observed stripe migration time; [O_2_] indicates the oxygen concentration in percentage.

**Fig 4:**
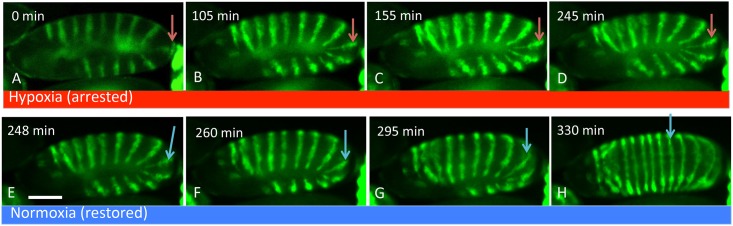
*engrailed* stripe migration was arrested under hypoxia and quickly resumed upon switching to normoxia. **(A)** An embryo with a fully developed *engrailed* pattern was positioned under hypoxia. **(B-D)** After 245 min of imaging, the stripe migration did not occur. **(E)** The oxygen condition was changed from hypoxia to normoxia. **(F)** The stripe migration was captured 12 min after the oxygen condition inside the device was changed. **(G, H)** The embryo had continuous stripe migration with normal speed. Images were taken every 3 min. Scale bar, 100 μm. Arrows indicate the 9^th^ stripe location under hypoxia (red) and normoxia (blue).

Notably, the arrested *engrailed* stripe migration due to a severe hypoxia can be rescued and resumed normally as soon as a normoxia condition is restored. A staged embryo was collected and positioned at a hypoxic region in the microfluidic device ([O_2_] <5%). Time-lapse imaging showed the *engrailed* stripe migration was paused for 240 min. At around 245 min, we quickly changed the oxygen condition to normoxia ([O_2_] = 20%). Clearly, the stripes started to migrate at a normal speed (Fig 4). These results showed that the embryo tail retraction was more sensitive to moderate and severe hypoxia. A compensating mechanism would put into action to maintain the necessities of life. Thus, the developmental activities were temporarily paused, until the normal oxygen condition is resumed.

### The effect of the oxygen gradient along the embryo AP axis

**Fig 5:**
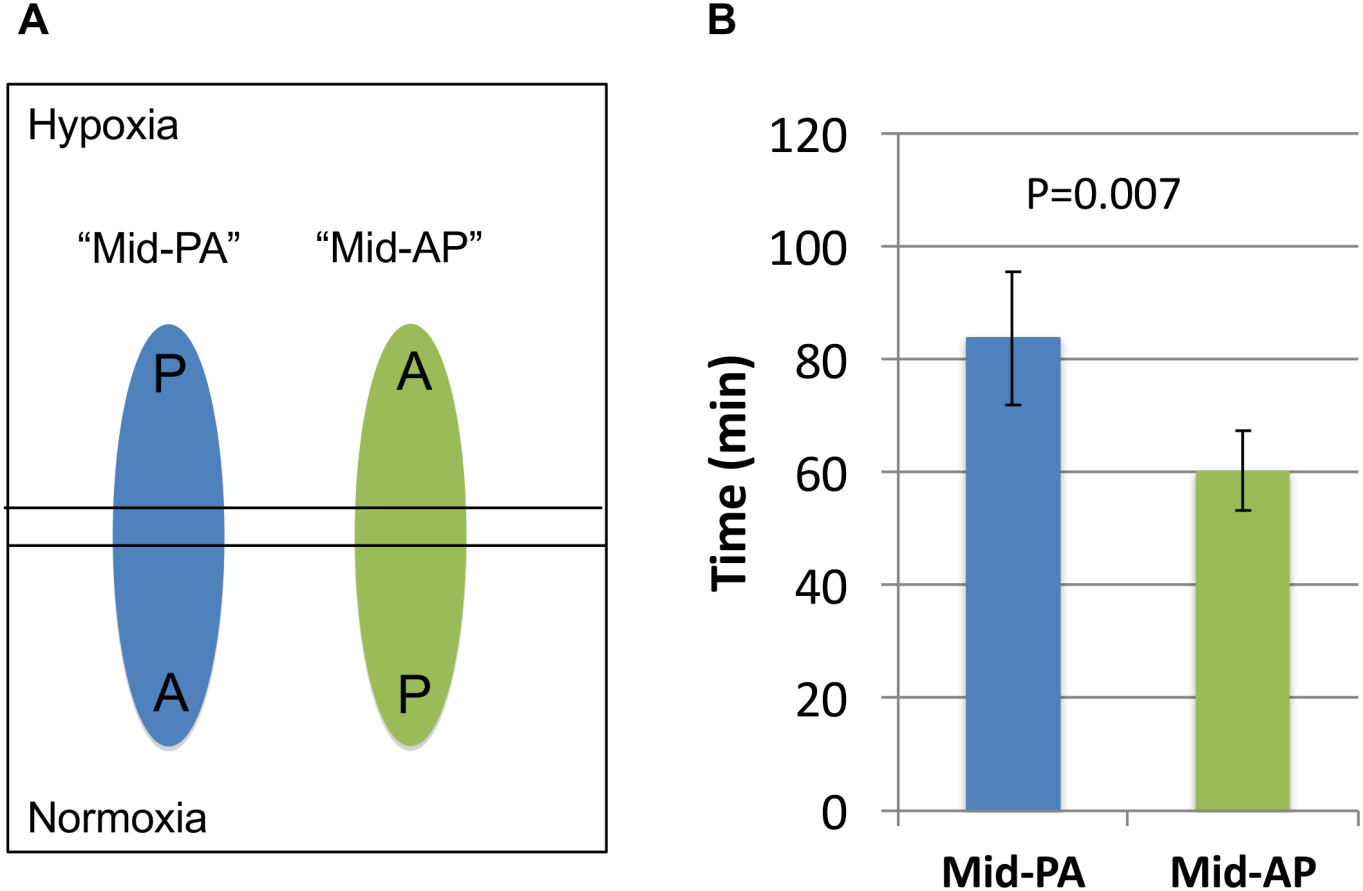
Different *engrailed* stripe migration time in A-P and P-A configuration allows the estimation of the permeability of oxygen though embryonic layers. **(A)** Two opposite geometries (mid-PA and mid-AP, respectively) in which embryos were positioned. A: Anterior, P: posterior. The microfluidic device center wall is outlined by two solid lines. The whole embryo bodies are under the same oxygen condition. However, the posterior ends in these two cases are under different oxygen condition locally. **(B)** When posterior was positioned close to hypoxic area, stripe migration took longer time than when positioned close to normoxic area, showing that the posterior is more sensitive to hypoxia during tail retraction.

Next, we investigated the effect of the oxygen gradient along the embryo AP axis on embryogenesis. Embryos were positioned with the AP axis perpendicular to the microchannels and across the center wall. Based on the embryo body length and oxygen concentration profile of the microfluidic device, an embryo could be exposed to an oxygen condition with the concentration ranging from approximately 7% to 14%. When an embryo was positioned reversely (e.g. from mid-AP to mid-PA), the oxygen conditions of the posterior and anterior would also be reversed accordingly (Fig 5A). Intriguingly, when the whole body of each embryo was positioned under the same oxygen concentration range, the “mid-PA” *engrailed* stripe migration time (83 ± 11 min) was significantly longer than the “mid-AP” time (60 ± 7 min, Fig 5B). Furthermore, the migration time in both cases were also very different from the previous results when the embryo’s posterior was positioned at the same location but had the body in parallel with the microchannels (A and P in the same oxygen condition). Knowing that the tail retraction was happening mainly at the posterior half of an embryo, when the posterior was close to the normoxia region (mid-AP), the averaged *engrailed* stripe migration time was 60 min: comparable to the time spent by the embryos that positioned in parallel with the microchannels in 10% oxygen. When the posterior was close to the hypoxia region (mid-PA), the migration time changed to 83 min on average, which was comparable to the time spent by the embryos that positioned in parallel with the microchannels in 8.3% oxygen. This is clearly indicating that the *engrailed* stripe migration not only responds to the environmental oxygen gradient locally at the tail, but is also affected by the current anterior oxygen condition. The whole embryo body has maintained a unique oxygen distribution by responding to the environmental oxygen conditions globally.

### The estimation of the oxygen permeability across embryonic layers

Since the embryo tail retraction time is closely correlated with the local oxygen concentration, the actual *engrailed* stripe migration time would indicate the true oxygen conditions in the tissue. This non-homogenous oxygen concentration profile inside the embryo body can be modeled using the oxygen profile outside the embryo and the relevant parameters: the oxygen permeability through the embryonic membrane, the oxygen diffusion rate inside the embryo, and the oxygen consumption rate of the cells. By simulating the oxygen concentration profile inside the embryo, based on all the parameters either measured or previously reported, the oxygen permeability across the embryonic membrane of live *Drosophila* embryos can be estimated.

To calculate the oxygen concentration profile inside an embryo, we established a model that described how oxygen permeated into the embryo across the embryo outer layers, diffused inside the embryo, and how it was consumed by the embryonic cells. When the embryo was positioned inside the microfluidic device with a stable oxygen gradient, the embryo’s trans-membrane oxygen perfusion, diffusion, and cellular consumption reached a dynamic equilibrium so that a steady state oxygen profile inside the embryo was established. We rebuilt the embryo body with the finite elements in 3D space. For each unit, a partial differential equation was made to describe the mass exchange based on Fick’s Law of diffusion. We assumed that the *engrailed* stripe migration time was directly reflecting the oxygen condition of the location where such activity occurred, and such condition was affected by the oxygen concentration both inside and outside the embryo. For example, if an embryo with an AP axis being perpendicular to the microchannels (A and P under different oxygen conditions) has the same stripe migration time as one with an AP axis in parallel with the microchannels (A and P under the same oxygen condition) does, we assume the average oxygen concentration at the tail region of these two embryos is the same. All functions were solved numerically. The oxygen diffusion coefficient inside the embryo was taken as $1\times 10^{-9}\left[\frac{m^{2}}{s}\right]$, which is the average of some of the previously reported values of similar cells and tissues. The cellular oxygen consumption rate was taken as $0.02\;\left[\frac{mol}{m^{3}\cdot s}\right]$ (see details in Table 1). Using our modeling methods, a simulated 3D oxygen concentration profile though the entire embryo body was created. In this 3D model, the oxygen gradients along both AP and VD axes were considered. Fitting the simulated oxygen profile inside the embryo to the measured values yields the previously unknown oxygen permeability across embryo membrane as 1×10^−3^ [cm/s] on average.

## Discussion

A previous report showed that *Drosophila* embryogenesis was affected when the embryos posterior and anterior halves were maintained at different temperatures [29]. In our study, by using a novel-designed microfluidic device, an oxygen gradient was provided to the living embryo on microscale. Our presumption was that *Drosophila* embryo’s developing rate would be affected in these altered oxygen conditions established within the microfluidic devices. It had been shown that, as another key environmental factor, the local oxygen concentration was also highly correlated with the embryogenesis. van Voorhies *et al* in their study showed that wild type flies metabolic rate was almost unaffected until the oxygen tension dropped to lower than 3%. The anoxic flies metabolic rate needed 30min to recover under normal condition [52]. Our results showed that several key embryo development processes were severely altered as the environmental oxygen condition changed: (1) delayed pole cell migration indicated the germ band extension reduced its speed by 4-fold at 5% oxygen; (2) Tail retraction was delayed as the oxygen supply became insufficient below 17% and could be fully arrested when oxygen concentration was below 5%. However, due to a severe hypoxia, the arrested tail retraction could be fully recovered shortly after normoxia was re-established with the normal developmental rate.

In the tracking of the *engrailed* strip migration, an exponential model was used to describe our findings and the nature of the embryo when responding environmental stress. Firstly, the negative time constant was indicating the stripe migration time delay as the oxygen concentration decreased. The best-fit curve described different levels of delay at various oxygen conditions. From our measurements, the *engrailed* stripe migration was more severely affected starting at 9% oxygen concentration, which could be a critical oxygen condition that triggered a compensating mechanism during germband shortening. Secondly, as mentioned in the results, the “500 min” point was also considered in the curve fitting, in order to best describe the embryo’s nature of the biophysical response to severe environmental stress. A plateau was seen from the extended exponential curve at about 5% of oxygen level (Fig 3B). This was indicating the cutoff oxygen level that the developmental activity could be paused at. Since none of the embryos showed any stripe migration at 5% oxygen or lower, the trend that the best-fit curve described was consistent with our observations.

The fluorescence tagged patterns we used, such as *engrailed* stripes and pole cell cluster, along with many other potential ones, were robust markers to track the morphological changes through the embryonic development stages. Using our methods, one can conveniently visualize and track the responding phenotypes under the changing environmental stress (e.g. oxygen concentration). We quantitatively described the response from the live embryo due to the insufficient oxygen supply. The critical oxygen concentration for the germband shortening was found. Our approaches provided a hint in understanding how complex tissue behaves in hypoxia.

By introducing an oxygen gradient along the AP axis during *Drosophila* embryogenesis, we can introduce unique oxygen conditions to different parts of the embryo body simultaneously. In nature, the environmental oxygen conditions may not vary in this manner for a *Drosophila* embryo. However, we believe that the microfluidics and live imaging methods we established here could potentially provide a powerful tool to study the individual developmental activity under spatiotemporally controlled hypoxia and the overall responses through the entire tissue body. Our approaches could also be further applied in understanding the dynamics of a complex system by providing a precise control of the environment. For the research methods that mainly rely on genetics and molecular approaches, our results provided a useful and complementary thought of seeking the information, e.g. the critical environmental conditions that trigger the compensation mechanisms starting to function and where the oxygen sensitive activities locate. However, due to the lack of molecular markers in our study, we were not able to track the subcellular level responses under environmental stress and the exact time point when compensation occurred. Our approach would also benefit from an improved microfluidic device design by considering the embryo size and oxygen gradient coverage even more precisely: it required a larger oxygen gradient, especially at the regions that are close to normoxia and anoxia, to provide a more distinguished oxygen condition that triggers the local oxygen sensitive activities in a fly embryo. This was part of the reason that, in our *engrailed* stripe migration time measurement, the embryo in <7% oxygen behaved very similarly.

Using the mathematical model, we could estimate the oxygen permeability in live *Drosophila* embryo, which otherwise can be challenging to quantify directly. When the values of oxygen consumption and diffusion rates changed, the estimated oxygen permeability value varied accordingly. For example, if oxygen consumption rate increased twice from our chosen number $0.02\;\left[\frac{mol}{m^{3}\cdot s}\right]$ to $0.04\;\left[\frac{mol}{m^{3}\cdot s}\right]$, the estimated permeability would only increase 100%. Additionally, when the diffusion rate changed from $1\times 10^{-5}\left[\frac{cm^{2}}{s}\right]\;$ to $2.1\times 10^{-5}\left[\frac{cm^{2}}{s}\right]\;$, the oxygen diffusion rate in pure water at room temperature [53], the estimated permeability would increase approximately 10-fold. Furthermore, based on the estimated oxygen permeability value, the inside oxygen condition can change from severe hypoxia to normoxia within 60 seconds following the outer oxygen profile, which is consistent with our data. This method yielded the first estimation of the oxygen permeability in live *Drosophila* embryos. The ability of providing a robust oxygen gradient on microscale could permit localized oxygen conditions, which are distinguished from part to part in a live tissue. This will help to understand the physiological behaviors and properties of a complex tissue under stress, while single cell model mainly focuses on examining the intracellular reactions. However, our model and results were based on several assumptions that lessened the real complexity of a living tissue. For example, in an embryo, different cell zones with specific functions may have different diffusion constants and the oxygen consumption rates are also known to change under varying external oxygen conditions. We only used constant values of these parameters to idealize the model and discussed the theoretical bases of indirectly obtain physical properties of a live tissue.

In this work, a microfluidic device was used to establish the oxygen concentration gradients on microscale, permitting the investigation of two important development processes during *Drosophila* embryogenesis. Our results showed that moderate hypoxia slowed down the embryogenesis (pole cells migration during the early embryogenesis and tail retraction during the middle embryogenesis). A severe hypoxia could arrest tail retraction, which could be fully rescued if a normoxia condition was re-established. Furthermore, oxygen permeability across live *Drosophila* embryonic layers was also estimated for the first time.

## Supporting information

S1 Movie *en* stripe migration at 17% oxygen. (AVI)

S2 Movie *en* stripe migration at 13% oxygen. (AVI)
